# Nonlethal Levels of Zeaxanthin Inhibit Cell Migration, Invasion, and Secretion of MMP-2 via NF-*κ*B Pathway in Cultured Human Uveal Melanoma Cells

**DOI:** 10.1155/2016/8734309

**Published:** 2016-01-28

**Authors:** Ming-Chao Bi, Nicole Hose, Cai-Lian Xu, Chen Zhang, Jodi Sassoon, E. Song

**Affiliations:** ^1^Department of Ophthalmology, The First Hospital of Jilin University, Changchun 130021, China; ^2^Tissue Culture Center, The New York Eye and Ear Infirmary of Mount Sinai, New York, NY 10003, USA; ^3^Fordham University, New York, NY 10023, USA; ^4^Shanghai Tenth People's Hospital, Tongji University School of Medicine, Shanghai 200072, China; ^5^Lixiang Eye Hospital of Soochow University, Suzhou 215021, China

## Abstract

Zeaxanthin at nonlethal dosages (3–10 *μ*M) significantly inhibited the cell migration of cultured uveal melanoma cells (C918 cell line) as determined by wound healing assay and Boyden chamber assay. Matrigel invasion assay showed that cell invasion of uveal melanoma cells could be significantly inhibited by zeaxanthin. Secretion of MMP-2 by melanoma cells was significantly inhibited by zeaxanthin in a dose-dependent manner as measured by ELISA kit. Zeaxanthin also significantly inhibited the NF-*κ*B levels in nuclear extracts of the UM cells, which is the upstream of the MMP-2 secretion. These results suggest that zeaxanthin might be a potentially therapeutic approach in the prevention of metastasis in uveal melanoma.

## 1. Introduction

Uveal melanoma (UM) is the most common intraocular malignant tumor in adults. UM has a high mortality rate due to a high incidence of metastasis that usually occurs in the liver [1–3].

Metastasis is the major cause of cancer-mediated death. Metastasis is a multistep process, which includes migration, adhesion, and invasion of cancer cells into the blood or lymphatic vessels that lead to the metastasis [4].

Matrix metalloproteinases (MMPs) are a group of proteolytic enzymes that play an important role in the degradation of extracellular matrix (ECM). MMP family members collectively can degrade all structural components of the ECM and lead to tumor cell migration, invasion, metastasis, and angiogenesis. The expression and activity of MMPs are increased in many types of human cancer, and this correlates with advanced tumor stage, increased invasion and metastasis, and shortened survival [4, 5]. MMP-2 is a member of the MMP family and can degrade matrix collagen and basement membrane. Overexpression of MMP-2 has been detected in various types of cancer. High levels of MMP-2 are associated with an increased of invasion and metastasis in several types of cancer [4–10].

Zeaxanthin, a natural bioactive, which belongs to the xanthophyll subclass of the carotenoid family, has been found to have specific cytotoxic effects on several types of cancer cells [11–18]. Our previous study revealed that zeaxanthin reduced the cell viability of UM cells whereas it did not affect the cell viability of normal ocular cells. Zeaxanthin induced apoptosis in human cultured UM cells through the activation of mitochondrial pathway [14]. However, to the best of our knowledge, the effects of zeaxanthin on the cell migration and invasion of UM cells have not been reported.

The purpose of the present study was to investigate the effects of nonlethal doses of zeaxanthin on the cell migration, invasion, and the secretion of MMP-2 by cultured human UM cells and its relevant signal pathways.

## 2. Material and Methods

### 2.1. UM Cell Lines

C918, a human choroidal melanoma cell line, was used in this study. C918 is an immortal UM cell line isolated from UM patients with metastasis by the University of Iowa. C918 was provided by Dr. Robert Folberg (University of Illinois, Chicago) and Dr. Xiaoliang Leon Xu (Memorial Sloan Kettering Cancer Center, New York). Cells were cultured with Dulbecco's modified Eagle's medium (DMEM) supplements with 10% fetal bovine serum [14].

### 2.2. MTT Assay

The viability of cells was determined by MTT assay that has previously been described [14]. Briefly, UM cells (5 × 10^3^/well) were seeded into 96-well plates and treated with zeaxanthin (0, 1, 3, 10, and 30 *μ*M) for 24 h. Zeaxanthin was obtained from Dr. Dennis L. Gierhart (Chesterfield, MO, USA) and was prepared as a stock solution at 60 mM by dissolving into DMSO (Sigma, St. Louis, MO, USA). After washing with medium, MTT (50 *μ*L of 1 mg/mL in DMEM) was used for the quantification of living cells. Mitochondrial dehydrogenases in living cells metabolize MTT to a purple formazan dye, which is measured photometrically at 540 nm by a microplate reader (Multiskan EX, Thermo, Ventana, Finland). Cell viability is proportional to the reading of absorbance and was expressed as the percentage of the reading from the control (cells cultured without zeaxanthin). All tests were performed in triplicate.

### 2.3. Wound Healing Assay

Cells were seeded in 12-well plates and were grown to nearly confluence. The cell monolayer was scratched with a 200 *μ*L micropipette tip to create a wound. The cultures were washed twice in PBS to remove float cells and debris and replaced with fresh culture medium. Cultures were photographed at various time periods by using an Olympus IX70 inverted phase-contrast microscope (Olympus Inc., Shinjuku-ku, Tokyo, Japan) [19]. Cells migrating from the leading edge were counted in 4 random fields and expressed as mean ± SD. The leading edge at different time periods was adjusted based on the width of unclosed wound of the control (cells not cultured with zeaxanthin). All tests were performed in triplicate.

### 2.4. Boyden Chamber Assay

Migration assays for UM cells were performed by using a 48-well Boyden chamber and a cellulose nitrate membrane with 8 *μ*m pore size (both from Neuro Probe, Inc., Gaithersburg, MD). The lower chamber was filled with DMEM with 10% serum. UM cells (2 × 10^4^ cells/well) in serum-free DMEM solution with or without zeaxanthin (10 *μ*M) were seeded into the upper chamber. After 8 h of incubation, cells on the upper surface of the membrane that had not migrated were gently scraped away with a cotton swab. The migrating cells on the lower side of the membrane were fixed with methanol and stained with hematoxylin. Ten fields were randomly selected and cells that had migrated to the lower surface of the membrane were counted under a light microscope at ×200 [20]. All tests were performed in triplicate.

### 2.5. Matrigel Invasion Assay

For cell invasion test, Corning Matrigel Invasion Chamber (8 *μ*m pore size, coated with Matrigel; Discovery Labware Inc., Bedford, MA, USA) was placed into the wells of 24-well culture plates. DMEM with 10% serum was added into the lower chamber; 5 × 10^4^ UM cells in serum-free DMEM with or without zeaxanthin (10 *μ*M) were added to the upper chamber and cultured. After 16 h of incubation, cells in the upper surface of the filter membrane that had not migrated were gently scraped away with a cotton swab. The invading cells on the lower surface of the filter membrane were fixed, stained, and counted as described above [20]. All tests were performed in triplicate.

### 2.6. Cell Culture for MMP-2 Secretion Assay

UM cells were seeded into 12-well plates and were grown to nearly confluence. The cultured medium was withdrawn and replaced with serum-free medium after washing the cells. Cells were cultured with or without zeaxanthin at various levels. After being cultured for 24 h, conditioned medium was collected and centrifuged at 500 ×g for 10 min, and the supernatants were collected and stored in vials at −70°C until analysis. All experiments were performed in triplicate.

### 2.7. Measurement of MMP-2 Protein

The protein amount of MMP-2 in the conditioned media was determined using the Human MMP-2 Quantitation ELISA kit (R&D Systems, Minneapolis, MN, USA) according to the manufacturer's instructions. Optical density was read by using a microplate reader at 450 nm and corrected with 540 nm. The amount of MMP-2 (pg/mL) was calculated from a standard curve and expressed as percentages of the negative controls (cells cultured without zeaxanthin). The sensitivity of the MMP-2 kit was 0.082 ng/mL.

### 2.8. Cell Culture for NF-*κ*B Assay

UM cells were plated into 6-well plates at a density of 1 × 10^6^ cells per well. After 24 h of incubation, the medium was replaced and cells were cultured with or without zeaxanthin (10 *μ*M) for 30 min. The culture medium was withdrawn and cultures were washed with cold PBS twice. Cells were scraped from the well and the nuclear fraction was extracted by using Nuclear Extraction Kit (BioSource, Camarillo, CA, USA). Cells were treated with Hypotonic Cell Lysis Buffer, incubated for 10 min at ice, treated with Detergent Solution, vortexed, and centrifuged (800 ×g, 6 min at 4°C). The pellet (nuclear fraction) was collected, washed with Nuclear Wash Buffer, and centrifuged (800 ×g, 6 min at 4°C). The pellet (nuclear fraction) was collected, treated with Complete Extraction Buffer, vortexed, incubated at ice, and centrifuged (14,000 ×g, 30 min at 4°C). The supernatants (nuclear extracts) were collected and stored at −70°C until analysis [21].

### 2.9. NF-*κ*B in Nuclear Extracts of Cultured UM Cells Assay

The amount of nuclear factor-kappa B (NF-*κ*B) in the nuclear extracts was measured by using NF-*κ*B ELISA kits (Invitrogen, Carlsbad, CA, USA) according to the manufacturer's instructions. The levels of NF-*κ*B were calculated using a standard curve and expressed as percentages of the negative controls (cells cultured without zeaxanthin). The sensitivity of this kit was <50 pg/mL. All tests were performed in triplicate [21].

### 2.10. Statistical Analysis

Data in each group were calculated and expressed as mean and standard deviation (mean ± SD). Statistical significances of difference of means throughout this study were calculated by Student's *t*-test in comparing data between two groups and ANOVA one-way test in comparing data from more than two groups. SPSS statistical software (SPSS Inc., Chicago, IL, USA) was used for the analysis of the data. A difference at *P* < 0.05 was considered to be statistically significant.

## 3. Results

### 3.1. Cell Viability Assay

**Figure 1:**
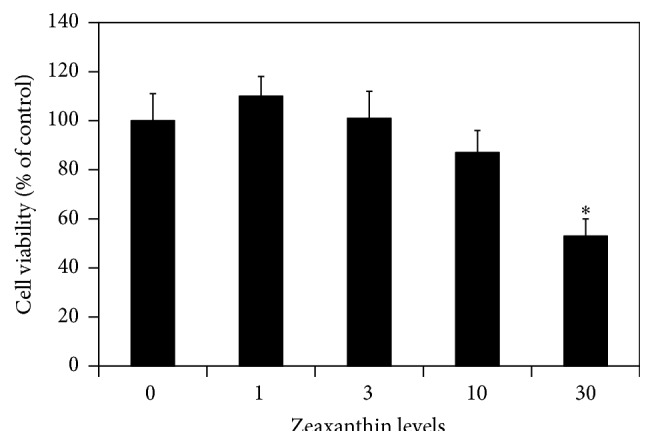
Effects of zeaxanthin on viability of uveal melanoma (UM) cells. Cultured human UM cells (C918) were seeded into 96-well plates and treated with zeaxanthin at different levels. MTT assay was used to determine the cell viability (see Material and Methods). Zeaxanthin only significantly affected the cell viability of UM cells (expressed as percentage of the controls) at 30 *μ*M (*n* = 3, *P* > 0.05). ^*∗*^ *P* < 0.05, versus control (cells cultured without zeaxanthin).

In MTT assay, zeaxanthin at the final levels of 1.0 and 3.0 *μ*M did not influence the cell viability of cultured human UM cells (*P* > 0.05, compared with cells not treated with zeaxanthin) (Figure 1). Cell viability in UM cells cultured with zeaxanthin at 10 *μ*M was slightly lower than that of the controls and the difference was statistically nonsignificant (*P* > 0.05), whereas cell viability was significantly lowered in 30 *μ*M zeaxanthin treated cells (*P* < 0.05). Dye exclusion staining showed that the number of nonvial cells was significantly increased in cells treated with 30 *μ*M zeaxanthin, but not in 1–10 *μ*M treated cells (data not shown). Therefore, level ranges 1–10 *μ*M of zeaxanthin were chosen as nonlethal dosages for subsequent experiments.

### 3.2. Wound Healing Assay

**Figure 2:**
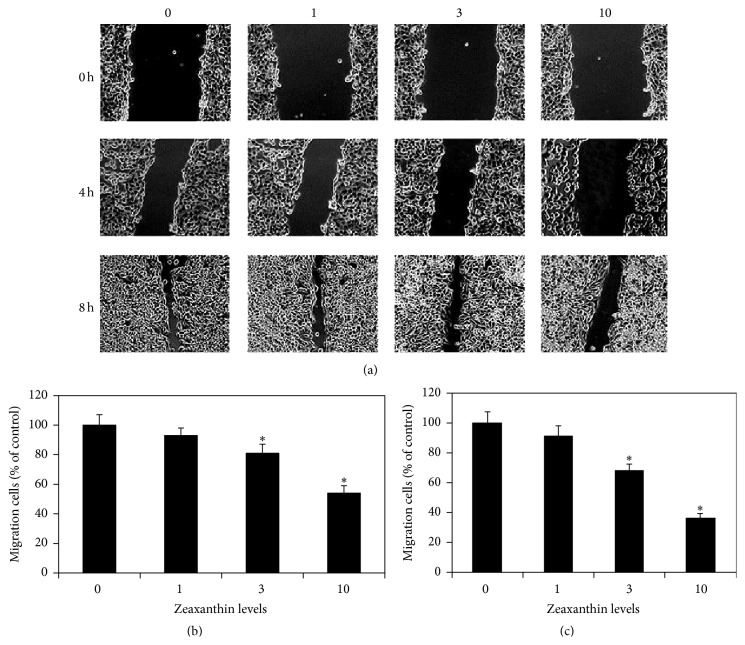
Zeaxanthin inhibits wound closure in cultured UM cells. Cultured human UM cells (C918) were seeded into 12-well plates, scratched, and treated with zeaxanthin (0, 1, 3, and 10 *μ*M) for 0, 4, and 8 h. Phase-contrast pictures of the wounds were taken for the comparison of wound closing process between cells treated with different levels of zeaxanthin at different periods (a). Cells migrating from the leading edge were counted at 4 h (b) and 8 h (c) and expressed as the percentage of the controls (cells cultured without zeaxanthin). After incubation for 4 h and 8 h, migrating cells in cultures treated with zeaxanthin at 3 and 10 *μ*M were significantly less than those from the controls (without zeaxanthin) (*P* < 0.05). Please see the text (Section 3.2) for the original data and the percentages of the controls (mean ± SD) of each group at different time periods.

UM cell cultures were scratched and cultured with 0, 1, 3, and 10 *μ*M zeaxanthin. Photos taken at 0, 4, and 8 h after scratch (Figure 2) show that the migration of UM cells was dose-dependently inhibited by zeaxanthin. After incubation for 4 h, cells migrating from the leading edge at cultures treated with zeaxanthin at 0, 1, 3, and 10 *μ*M were 152.5 ± 10.2, 142.0 ± 7.5, 124.0 ± 9.3, and 82.0 ± 6.6 (mean ± SD), respectively, and expressed as the percentages of the controls (without zeaxanthin) at 1.00 ± 0.07, 0.93 ± 0.05, 0.81 ± 0.06, and 0.54 ± 0.05 (mean ± SD), respectively (Figure 2(b)). Migrating cells in cells cultured with 1 *μ*M zeaxanthin were not significantly different from those in the controls (cells cultured without zeaxanthin) (*P* > 0.05). Migrating cells at 3 and 10 *μ*M zeaxanthin groups were significantly less than those in the controls and cultured with 1 *μ*M zeaxanthin (*P* < 0.05). Migrating cells at 10 *μ*M zeaxanthin group were significantly less than those cultured with 3 *μ*M zeaxanthin (*P* < 0.05). After incubation for 8 h, cells migrating from the leading edge at cultures treated with zeaxanthin at 0, 1, 3, and 10 *μ*M zeaxanthin were 196.8 ± 12.8, 178.5 ± 13.2, 134.5 ± 8.8, and 70.3 ± 6.8, respectively, and expressed as the percentage of the controls at 1.00 ± 0.07, 0.91 ± 0.07, 0.68 ± 0.04, and 0.36 ± 0.03, respectively (Figure 2(c)). Migrating cells in cells cultured with 1 *μ*M zeaxanthin were not significantly different from those in the controls (*P* > 0.05). Migrating cells at 3 and 10 *μ*M zeaxanthin groups were significantly less than those in the controls and with 1 *μ*M zeaxanthin (*P* < 0.05). Migrating cells at 10 *μ*M zeaxanthin group were significantly less than those cultured with 3 *μ*M zeaxanthin (*P* < 0.05) (Figure 2).

### 3.3. Boyden Chamber Assay

**Figure 3:**
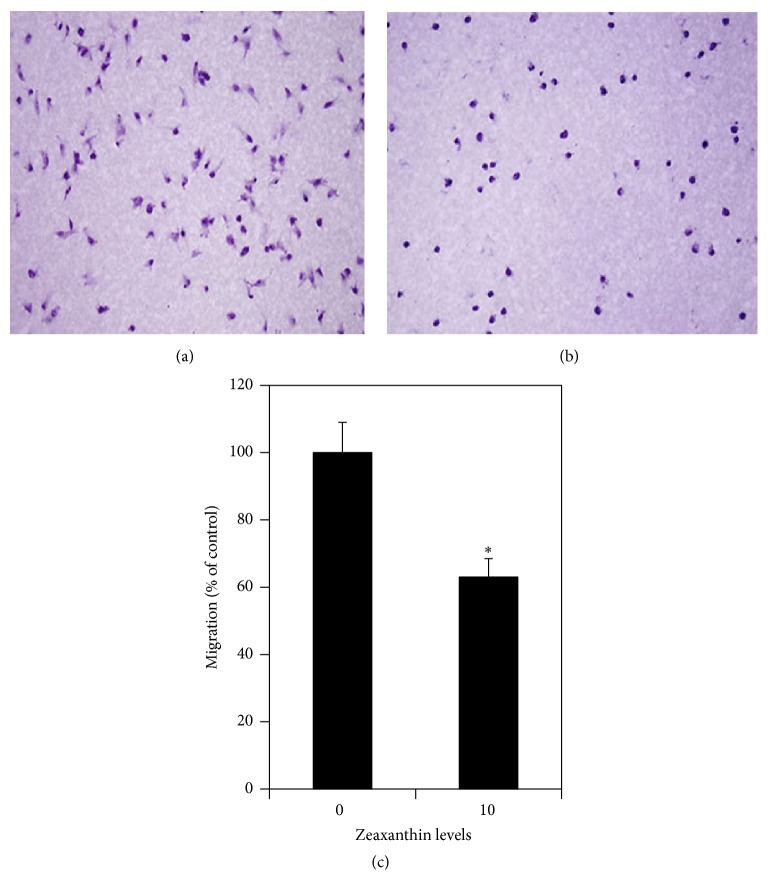
Zeaxanthin inhibits migration of UM cells by using Boyden chamber assay. Cells (C918) were seeded into the upper chamber and the lower chamber was filled with DMEM with 10% serum (see Material and Methods). After being cultured with (b) or without (a) zeaxanthin at 10 *μ*M for 8 h, the migration of UM cells was measured by counting the migrating cells on the lower surface of the membrane at 10 fields. Photos were taken by a light microscope at ×200. Numbers of migrating cells in cultures without zeaxanthin and with zeaxanthin were 107.3 ± 9.75 and 67.2 ± 6.16 cells (mean ± SD), respectively, and expressed at the bar graph as 1.00 ± 0.09 and 0.63 ± 0.06 (percentages of the control), respectively. Zeaxanthin significantly inhibited the migration of UM cells (*n* = 3, *P* < 0.05) (c). ^*∗*^ *P* < 0.05, versus control (cells cultured without zeaxanthin).

The effects of zeaxanthin on UM cell migration were studied by Boyden chamber assay. Cells were treated with and without zeaxanthin (10 *μ*M) and cultured for 8 h. The results are shown in Figures 3(a), 3(b), and 3(c). Numbers of migrating cells in cultures with and without zeaxanthin were 67.2 ± 6.16 and 107.3 ± 9.75 cells, respectively. Zeaxanthin significantly decreased the transmembrane migration of UM cells as compared with cells not treated with zeaxanthin (*P* < 0.05).

### 3.4. Matrigel Invasion Assay

**Figure 4:**
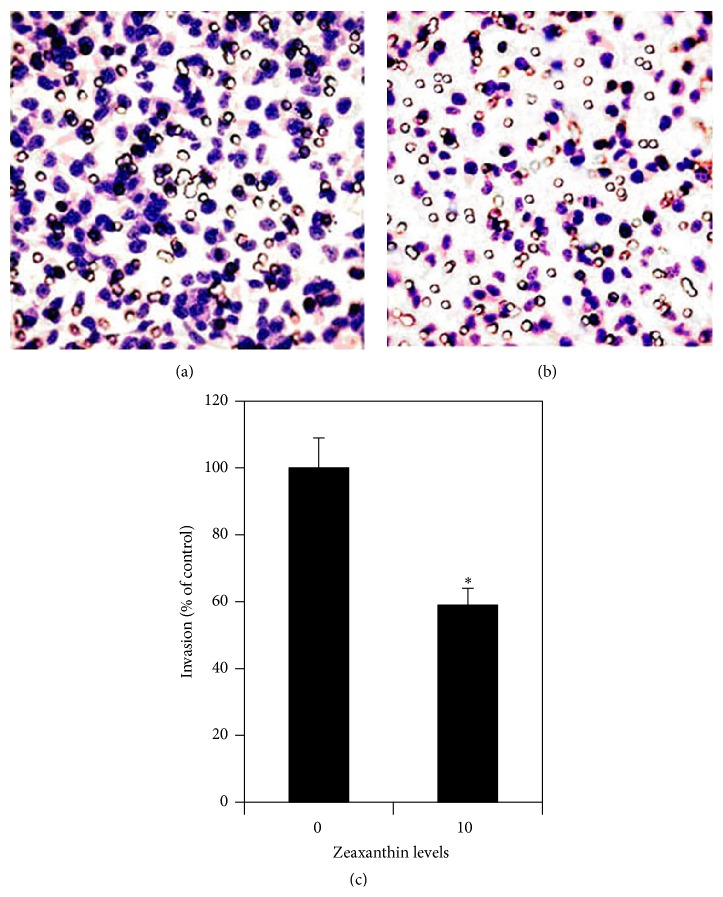
Zeaxanthin inhibits invasion of UM cells by using Matrigel invasion assay. Cells (C918) were seeded into the Matrigel Invasion Chamber and placed into a 24-well plate with DMEM supplemented with 10% serum. After being cultured with (b) or without (a) zeaxanthin at 10 *μ*M for 16 h, the invasion of UM cells was measured by counting the invading cells on the lower surface of the filter membrane at 10 fields. Numbers of invading cells in cultures without zeaxanthin and with zeaxanthin were 231.9 ± 20.4 and 135.8 ± 12.2 cells (mean ± SD), respectively, and expressed at the bar graph as 1.00 ± 0.09 and 0.59 ± 0.05 (percentages of the control), respectively. Photos were taken by a light microscope at ×200. Zeaxanthin significantly inhibited the invasion of UM cells (*n* = 3, *P* < 0.05) (c). ^*∗*^ *P* < 0.05, versus control (cells cultured without zeaxanthin).

The effects of zeaxanthin on cell invasion of UM cells were studied by Matrigel Invasion Chamber. Cells were treated with and without zeaxanthin (10 *μ*M) and cultured for 16 h. The results are shown in Figures 4(a), 4(b), and 4(c). Numbers of invaded cells in cultures with and without zeaxanthin were 135.8 ± 12.2 and 231.9 ± 20.4 cells, respectively. Zeaxanthin significantly decreased the invasion of UM cells as compared with cells not treated with zeaxanthin (*P* < 0.05).

### 3.5. Secreted MMP-2 Protein Assay

**Figure 5:**
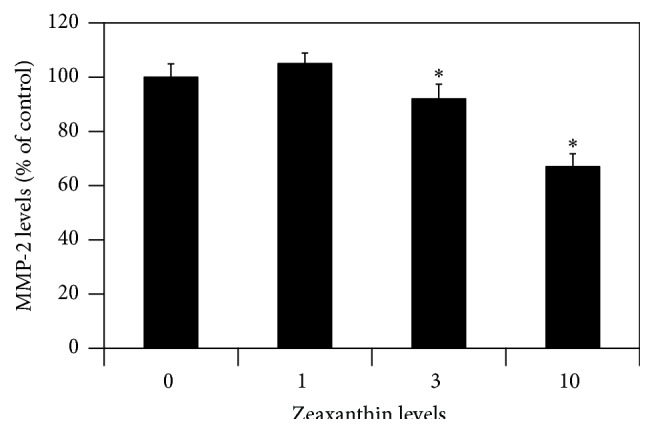
Zeaxanthin inhibits secretion of MMP-2 protein by cultured UM cells. UM cells were seeded into 12-well plates and were cultured with or without zeaxanthin (10 *μ*M). After being cultured for 24 h, conditioned medium was collected and centrifuged and the supernatants were collected. The amount of MMP-2 in the supernatants was determined using the Human MMP-2 ELISA kit. Zeaxanthin at 3 *μ*M and 10 *μ*M significantly inhibited the secretion of MMP-2 (*n* = 3, *P* < 0.05). ^*∗*^ *P* < 0.05, versus control (cells cultured without zeaxanthin).

One-way ANOVA analysis of the results on the MMP-2 assay revealed that zeaxanthin had a dose-dependent inhibitory effect on the secretion of MMP-2 protein by UM cells (*P* < 0.05) (Figure 5). Secretion of MMP-2 by UM cells treated with zeaxanthin at 3.0 and 10.0 *μ*M was significantly less than that from the negative control (cells not treated with zeaxanthin, *P* < 0.05).

### 3.6. NF-*κ*B in Nuclear Extracts of Cultured UM Cells Assay

Zeaxanthin (10.0 *μ*M) treatment decreased NF-*κ*B levels in nuclear extracts of the UM cells. The levels of NF-*κ*B in nuclear extracts in cells cultured with zeaxanthin were only 42% of the control values (cells not treated with zeaxanthin). The difference of NF-*κ*B levels between cells treated with and without zeaxanthin was statistically significant (*P* < 0.05).

## 4. Discussion

In the present study, the nonlethal dosages of zeaxanthin significantly inhibited the cell migration of cultured human UM cells as demonstrated by the wound healing assay and the Boyden migration assay.

Matrigel is the extracellular matrix secreted by the Engelbrecht-Holm-Swarm mouse sarcoma cell line. It contains laminin, collagen IV, nidogen/entactin, and proteoglycans and resembles the basement membrane [4]. Cell invasion is usually tested by the use of Matrigel Invasion Chamber. In the present study, zeaxanthin significantly inhibited the invasion of UM cells through the Matrigel-coated membrane from the upper surface of the membrane to the lower side.

MMPs are a group of zinc-dependent proteinase capable of digesting virtually any component of the ECM to enhance the migration, invasion, and metastasis of cancer cells. The MMPs could be divided into collagenases, gelatinases, stromelysins, and matrilysins on the basis of their specificity for ECM components or be grouped according to their structure. MMP-2 (also called 72-kDa type IV collagenase, or gelatinase A) belongs to gelatinases (based on the substrate) or gelatin binding MMP group (based on the structure) [4]. MMP-2 can degrade denatured collagen (gelatin), native collagens IV, V, VI, and X, elastin, and fibronectin [4, 22]. It has been reported that MMP-2 also degrades native collagen I, the main component of mammal scleral protein [22].

MMP-2 has been detected in the UM pathologic specimens and cell lines [23–29]. UM cell lines from metastasis patients show a higher level of MMP-2 [29]. MMP-2 expression is associated with higher incidence of metastatic diseases and lower survival rate [23, 24]. It has been reported that MMP-2 may be used as a prognostic marker in UM [24].

In the present study, zeaxanthin dose-dependently inhibited the secretion of MMP-2 by UM cells. The inhibitory effects of zeaxanthin on the secretion of MMP-2 by UM cells might cause the inhibition of cell migration and invasion of UM cells by zeaxanthin. This is consistent with previous reports that various medications can inhibit the cell migration and invasion of various cancer cells through the inhibition of MMP-2 [19, 20, 30].

NF-*κ*B is a major transcription factor that promotes the expression of many genes involved in a variety of cellular processes [31]. NF-*κ*B is present in the cytoplasm in an inactive NF-*κ*B complex which could be activated by various stimuli. Activated NF-*κ*B translocates to the nucleus and binds to the promoter or enhancer regions of specific genes and then induces the expression of relevant genes, including various MMPs [31, 32].

NF-*κ*B is constitutively activated in UM cells [32]. The expression of NF-*κ*B in metastatic UM is higher than that of the primary tumor [33, 34]. NF-*κ*B inhibitor BAY11-7082 markedly decreased the nuclear translocation of NF-*κ*B and inhibits the migration of human UM cells [32]. miR-9 is significantly reduced in highly invasive UM cell lines. miR-9 suppresses UM cell migration and invasion through downregulation of NF-*κ*B signaling pathway [35].

In the present study, zeaxanthin inhibited the secretion of MMP-2 protein and decreased NF-*κ*B levels in nuclear extracts of the UM cells, suggesting that zeaxanthin inhibits the secretion of MMP-2 via NF-*κ*B signal pathway. This is consistent with the previous reports that NF-*κ*B is the upstream of MMP-2 in various cancer cells [20, 32, 35–37].

Recently, Xu et al. published their studies regarding the effects of zeaxanthin on the growth and invasion of UM in nude mice eyes and revealed that zeaxanthin significantly inhibited the invasion of uveal melanoma [38]. This in vivo invasion inhibitory effect of zeaxanthin on UM was consistent with the result of our in vitro study. Furthermore, Wu et al. found that fibroblasts cultured with cutaneous melanoma conditioned medium showed an increase of migration. Zeaxanthin inhibited melanoma-induced fibroblast migration [39]. This report indicated that zeaxanthin not only influenced the migration of UM as revealed by us but also inhibited factors secreted by cutaneous melanoma that stimulates the migration of fibroblast.

In conclusion, this study demonstrated that, in addition to the previously reported zeaxanthin-induced apoptosis effects on UM cells, zeaxanthin can also inhibit the cell migration and invasion of cultured human UM cells by the decrease of secretion of MMP-2. This effect is attributed to the inhibition of NF-*κ*B pathway in UM cells by zeaxanthin. The results of this study further suggest that zeaxanthin might be a potentially therapeutic approach in the management of uveal melanoma.
